# Rapid and gradual modes of aerosol trace metal dissolution in seawater

**DOI:** 10.3389/fmicb.2014.00794

**Published:** 2015-01-21

**Authors:** Katherine R. M. Mackey, Chia-Te Chien, Anton F. Post, Mak A. Saito, Adina Paytan

**Affiliations:** ^1^Earth System Science, University of California, IrvineIrvine, CA, USA; ^2^Department of Earth and Planetary Sciences, University of California, Santa CruzSanta Cruz, CA, USA; ^3^Graduate School of Oceanography, University of Rhode IslandNarragansett, RI, USA; ^4^Marine Chemistry and Geochemistry, Woods Hole Oceanographic InstitutionWoods Hole, MA, USA; ^5^Institute for Marine Science, University of California, Santa CruzSanta Cruz, CA, USA

**Keywords:** aerosols, atmospheric deposition, phytoplankton, trace metals, ligands

## Abstract

Atmospheric deposition is a major source of trace metals in marine surface waters and supplies vital micronutrients to phytoplankton, yet measured aerosol trace metal solubility values are operationally defined, and there are relatively few multi-element studies on aerosol-metal solubility in seawater. Here we measure the solubility of aluminum (Al), cadmium (Cd), cobalt (Co), copper (Cu), iron (Fe), manganese (Mn), nickel (Ni), lead (Pb), and zinc (Zn) from natural aerosol samples in seawater over a 7 days period to (1) evaluate the role of extraction time in trace metal dissolution behavior and (2) explore how the individual dissolution patterns could influence biota. Dissolution behavior occurs over a continuum ranging from rapid dissolution, in which the majority of soluble metal dissolved immediately upon seawater exposure (Cd and Co in our samples), to gradual dissolution, where metals dissolved slowly over time (Zn, Mn, Cu, and Al in our samples). Additionally, dissolution affected by interactions with particles was observed in which a decline in soluble metal concentration over time occurred (Fe and Pb in our samples). Natural variability in aerosol chemistry between samples can cause metals to display different dissolution kinetics in different samples, and this was particularly evident for Ni, for which samples showed a broad range of dissolution rates. The elemental molar ratio of metals in the bulk aerosols was 23,189Fe: 22,651Al: 445Mn: 348Zn: 71Cu: 48Ni: 23Pb: 9Co: 1Cd, whereas the seawater soluble molar ratio after 7 days of leaching was 11Fe: 620Al: 205Mn: 240Zn: 20Cu: 14Ni: 9Pb: 2Co: 1Cd. The different kinetics and ratios of aerosol metal dissolution have implications for phytoplankton nutrition, and highlight the need for unified extraction protocols that simulate aerosol metal dissolution in the surface ocean.

## Introduction

The role of atmospheric nutrient deposition in supporting marine phytoplankton growth is well documented (Peierls and Paerl, 1997; Herut et al., 1999; Mills et al., 2004; Duce et al., 2008; Mackey et al., 2012a,c). Determination of bioavailable nutrient and trace metal content in aerosols is based on laboratory extraction methods that generate operationally-defined solubility patterns sensitive to extraction volume, solvent pH, mechanical agitation, and other factors. In this study, we investigate the role of extraction time on aerosol leaching kinetics, with the goal of characterizing the dissolution chemistry of a suite of trace metals (Al, Cd, Co, Cu, Fe, Mn, Ni, Pb, and Zn) and understanding the impact of these characteristics on marine biota.

Atmospheric deposition of nitrogen (N) and phosphorus (P) provide nutrition in open ocean (Duce et al., 2008; Mackey et al., 2012a,c) and coastal areas (Paerl, 1997; Peierls and Paerl, 1997; Herut et al., 1999), and atmospheric N deposition may supply 40–70% of the total nitrate to phytoplankton in the North Pacific Ocean (Prospero and Savoie, 1989). Phytoplankton growth can be stimulated by these macronutrient additions as demonstrated in mesocosm (Guieu et al., 2010, 2014; Laghdass et al., 2011; Giovagnetti et al., 2013; Ridame et al., 2013; Wuttig et al., 2013c) and shipboard incubation experiments (Mills et al., 2004; Davey et al., 2008; Langlois et al., 2012). Deposition of iron (Fe) is another important source of growth-sustaining nutrition for phytoplankton, and atmospheric deposition is a primary source of Fe to many regions of the ocean (Duce and Tindale, 1991; Mills et al., 2004; Mahowald et al., 2005; Moore et al., 2006; Baker and Croot, 2010).

In recent years the biological effects of metals other than Fe derived from atmospheric deposition have received increasing attention (Jordi et al., 2012; Mackey et al., 2012a; Wuttig et al., 2013c). Biologically relevant metals such as Cd, Co, Cu, Mn, Ni, and Zn are cofactors in a wide range of microbial enzymes (Morel et al., 1994; Morel and Price, 2003 and references therein; Saito et al., 2011). Metal micronutrients can limit or co-limit phytoplankton growth (Saito et al., 2002; Saito and Goepfert, 2008); however, in high concentrations metals can cause toxicity (Paytan et al., 2009; Jordi et al., 2012). Biological metal uptake is also apparent for certain metals from depth profiles even in areas where metals do not necessarily limit growth (e.g., Zn and Co, Saito and Moffett, 2002; Noble et al., 2008), whereas the profiles of other metals are more strongly influenced by abiotic factors despite their critical biological roles (e.g., Mn, Noble et al., 2008). Similar to Fe, input of metals from atmospheric deposition of crustal and anthropogenic particles may be the main source of these metals to certain regions, particularly the open ocean where other geological sources (rivers, groundwater) are minor. Phytoplankton growth responses to seawater soluble metals from atmospheric deposition has been observed following natural (Jordi et al., 2012) and simulated (Mackey et al., 2012a) deposition events.

Because investigation of the biological role of metal deposition is still relatively new, much of our predictive capacity comes from atmospheric deposition models. By making assumptions about deposition rates, total metal content, and metal solubility, these models predict the amount of metal that will dissolve in seawater following deposition on the ocean's surface. For these models the concept of “solubility” and “fractional solubility” are important. Baker and Croot (2010) define metal solubility as being operationally determined by measuring metal concentrations in filtrate after passing through a filter containing the aerosol sample. Fractional solubility is defined as the portion of metal that dissolves from an aerosol sample, and is determined from the metal content in the filtrate divided by the total metal content of the aerosol added to the solution (Baker and Croot, 2010). Both of these parameters are operationally defined and sensitive to experimental protocols.

Aerosol leaching procedures differ considerably from conditions in the surface ocean. Metal solubility estimates are typically made by extracting aerosol samples under controlled conditions using pure water or buffered acidic solvents that approximate rain water (Anderson et al., 2010 and references therein). At the same time a number of factors can influence metal dissolution during experiments. Different aerosol mineralogical properties and atmospheric particle processing strongly influences aerosol metal solubility (Desboeufs et al., 1999, 2001, 2003, 2005; Jones and Gislason, 2008; Journet et al., 2008; Paris et al., 2010, 2011; Paris and Desboeufs, 2013).

The composition and history of the seawater used in dissolution experiments has also been found to influence the solubility of Fe and other elements. For example, seawater superoxide and hydrogen peroxide levels (Heller and Croot, 2011; Wuttig et al., 2013a,b) as well as ligand content (Saito et al., 2005; Mackey et al., 2012a) exert control on seawater metal chemistry, potentially influencing the dissolution of metals from aerosols. The solvent used in a dissolution experiment likewise influences solubility measurements made in the laboratory (see Table 1 for chemistry of seawater used in this study). Chen et al. (2006) directly compared the solubility of a suite of metals in aerosol samples extracted in pure water and seawater. While certain metals, such as Zn, had similar solubility in the two solvents, others metals like Fe and Al were much less soluble in seawater than in pure water.

**Table 1:** **Concentrations of trace metals in seawater used in the leaching experiments**.

**Metal**	**Background seawater concentration, this study (ng/L)**	**Background seawater concentration, this study (nmol/L)**	Coefficient of variation (%)^*^	**Typical atlantic ocean surface concentration (nmol/L)**
Al	517	19.2	21	33^a^
Cd	12	0.11	6	0.014^b^
Co	0.55	0.0093	21	0.017^c^
Cu	30	0.47	11	1.15^d^
Fe	DL	DL	19	0.08^b^
Mn	32	0.58	7	0.76^e^
Ni	141	2.4	10	2.05^d^
Pb	2.2	0.012	3	0.05^f^
Zn	318	4.86	30	0.04^d^

*DL, below detection limit*.

* *determined from time zero measurement of 4 seawater-only operational blank replicates*.

a (Hydes, 1983);

b (Martin et al., 1993);

c (Saito and Moffett, 2002);

d (Bruland and Franks, 1983);

e (Statham et al., 1998);

f *(Boyle et al., 2014)*.

Other studies have directly investigated Fe solubility in seawater (Zhuang et al., 1990; Zhu et al., 1997; Bonnet and Guieu, 2004; Buck et al., 2006, 2013), however in these studies the duration and degree of mechanical agitation have all varied, thus it is difficult to compare the results. For example, up to 60% of the variability in trace metal solubility can be attributed to the type of filter used to collect the aerosol samples (Buck and Paytan, 2012). Difficulty comparing across data sets limits the accuracy of biogeochemical models that incorporate these data. Boyd et al. (2010) conducted an extensive review of aerosol Fe solubility measurements and biogeochemical modeling approaches for atmospheric Fe deposition. They conclude that uncertainty in Fe solubility estimates and model parameters have led to an overstatement of the relationship between dust supply and biological response, and argue that to improve biogeochemical model accuracy for Fe deposition it is necessary to standardize dissolution methodologies.

**Figure 1:**
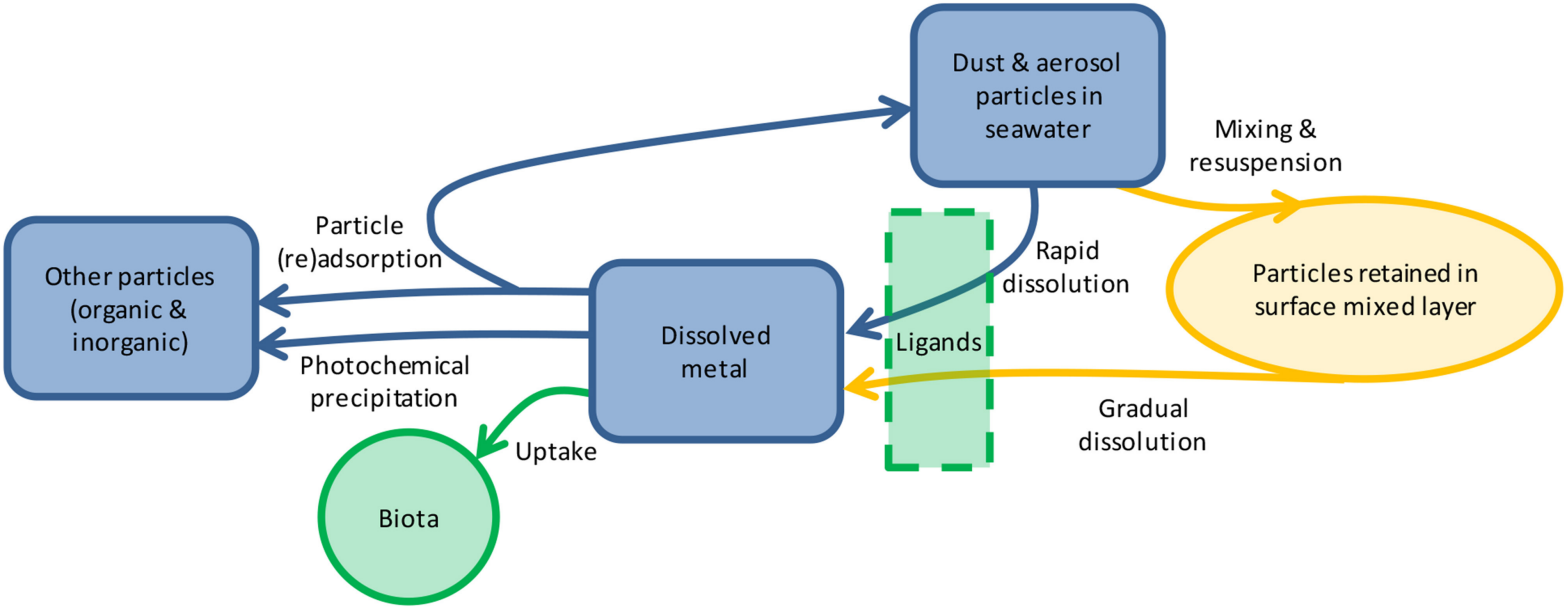
**Schematic showing the reservoirs (shapes) and processes (arrows) influencing aerosol metal dissolution in the surface ocean**. Once particles are deposited through atmospheric deposition, metals will either dissolve rapidly or gradually over time as they are mixed within the surface ocean. Dissolution may be mediated by ligands, depending on the metal. Dissolved metals may be taken up by biota, such as phytoplankton and microbes, undergo photochemical transformations, or (re)adsorb onto particles. The relative importance of each process varies for each metal depending on its chemical characteristics, biological role, and particle reactivity.

In natural waters, trace metal solubility is affected by aerosol properties (particle size, composition, source, transport distance, etc.), seawater chemistry, biological ligand concentration, and photochemistry (Figure 1; Barbeau et al., 2001; Aguilar-Islas et al., 2010; Measures et al., 2010; Buck et al., 2013). Less is known about how the duration of particle interaction with the solution affects the dissolution of metals from aerosols. The residence time of particulate metals in the upper water column ranges from days to months [e.g., particulate Fe is 6–62 days (Croot et al., 2004) and particulate Al is 3–22 days (Dammshäuser et al., 2013)], yet many leaching protocols occur over seconds to hours (Buck et al., 2006; Chen et al., 2006). Longer leaching time has been shown to increase the dissolution of P (Ridame and Guieu, 2002; Mackey et al., 2012c) and some metals in seawater (e.g., Al, Co Fe, Mn, Zn; Boyd et al., 2010; Measures et al., 2010; Mendez et al., 2010; Thuroczy et al., 2010); however, information on dissolution of many biologically important metals from the same samples using the same extraction procedures is needed. In this study we investigated the dissolution behavior of nine aerosol metals in seawater over a 7 days time course. We found that metal dissolution behavior falls along a continuum and is characterized by significant inter-sample variability, with important ecological and modeling implications.

## Materials and methods

### Aerosol collection

Samples were collected with a Total Suspended Particle (TSP) High Volume Sampler (HVS) at the shoreline of the Gulf of Aqaba, Red Sea at the Inter-University Institute for Marine Science (29°31′N, 34°55′E) as described in Chen et al. (2006). Samples were collected over 3 days periods at a flow rate of 1.2–1.5 m^3^ h^−1^, and approximately 200 m^3^ of air was filtered. The sampler collects all particles greater than 0.2 μm and does not have an upper limit size cutoff. The particle size distributions are given in Table 2, and were made using a GT-321 Handheld Particle Counter. The size classes included 0.3, 0.5, 1, 3, and 5 μm. Samples were collected on acid-cleaned, milliQ water-rinsed 47 mm polycarbonate membrane filters (Isopore) and sealed frozen in plastic petri dishes until use. Three replicate filters were collected over each 3 day sampling period, and duplicate filters from the same sampling period were combined in the dissolution experiment described below. The third filter was used to determine total metal content in the aerosol particles following heated digestion with nitric and hydrofluoric acid as described in Chen et al. (2006). Filters were weighed before and after sample collection and were totally dry when weighed. Aerosol mass was determined by subtraction and the precision was 0.01 mg.

**Table 2:** **Aerosol properties. Particle size ranges are the average of 10 measurements by the particle counter**.

**Aerosol collection start date**	**Aerosol collection end date**	**Total suspended particle concentration (μg/m3)**	**Total aerosol mass (mg)**	**≥ 5 um (particles/L)**	**≥ 2 um (particles/L)**	**≥ 1 um (particles/L)**	**≥ 0.5 um (particles/L)**	**≥ 0.3 um (particles/L)**
2/26/2006	3/1/2006	22	12.6	186	1401	2356	5960	86403
5/7/2006	5/10/2006	77	9.1	91	854	2127	13404	177820
6/11/2006	6/14/2006	43	6.7	280	2327	4075	7437	63767
6/15/2006	6/18/2006	44	8.0	167	2676	5077	10870	153522
7/9/2006	7/12/2006	29	5.2	ND	ND	1705	3905	60958
3/12/2006	3/15/2006	97	9.8	317	2780	4421	8045	37915
3/12/2006	3/15/2006	97	6.6	317	2780	4421	8045	37915

*ND = not determined*.

### Dissolution experiment

To determine the effect of extended seawater contact on aerosol trace metal dissolution, experiments were performed with natural TSP samples in low nutrient, low metal seawater collected from the South Atlantic Ocean (13.480 S 0.040 W, 8 m depth, salinity 36.4). Seawater was aged over 1 year at room temperature in a polyethylene carboy under trace metal clean conditions in the dark and 0.2 μm filtered immediately before use. Six distinct TSP samples on whole filters were added separately to 300 mL of seawater in 500 mL 10% hydrochloric acid cleaned polycarbonate bottles and incubated in the dark on a rotary shaker on medium speed. Two operational blanks were included using acid cleaned filters rather than true samples, and were incubated, sampled, and analyzed identically to the real samples. At each time point the bottles were shaken and 13 mL aliquots were removed by pouring from the bottle into freshly acid cleaned syringes, filtered through 0.2 μm polypropylene syringe filters, and stored in 15 mL centrifuge tubes at room temperature until analysis. All sampling was done in a class 100 clean room within a laminar flow hood equipped with HEPA filter and no metal parts or metal equipment. Sampling time points occurred immediately following sample addition (within 10 min) and then after 6 h, 1 day, 3 days, and 7 days of soaking. Blank seawater containing no aerosol was also collected at time zero to determine the background concentration of metals.

The particle to solvent ratio is known to affect solubility of nutrients and trace metals from aerosols, where higher particle loads can reduce the solubility of certain metals due to re-adsorption onto particle surfaces (Baker et al., 2006). Differences in particle load existed in this experiment (Table 2). To determine the effect of particle load on metal dissolution in this study, we incubated one sample at two particle concentrations. For this sample (3/12/2006) one bottle received 9.8 mg of sample and another bottle received 6.6 mg of sample, and both were added to 300 mL of seawater.

This aerosol loading is on the high end of values for similar metal leaching studies. Natural aerosol loadings in the ocean vary by site depending on the TSP concentration in air and the depositional velocity. For example, in areas of relatively high deposition such as near Cape Verde and in the Gulf of Aqaba, aerosol loadings of 1–2 mg L^−1^ have been employed (Paytan et al., 2009; Heller and Croot, 2011). The aerosol loadings used here were higher (1742 mg L^−1^) compared to prior published datasets where values from 10 to 100 mg L^−1^ were used (Chen et al., 2006; Buck and Paytan, 2012).

### Trace metal column chemistry and analysis

Seawater samples from the dissolution experiment were acidified to pH <2.0 with concentrated trace metal grade nitric acid (final concentration 0.02 M) at least 48 h before removal from their storage tubes. The pH was then adjusted to six with ammonium acetate (final concentration 0.05 M) and ammonium hydroxide (final concentration 0.027 M) prior to column chemistry. Trace metals were concentrated using Nobias Chelate-PA1 resin (HITACHI High Technologies, Japan) to remove the seawater matrix (Sohrin et al., 2008; Biller and Bruland, 2012). Metals were eluted with 1 M trace metal grade nitric acid; method detection limits and recovery are given in the online Supplemental Material. The 5 mL eluent from each sample was analyzed for Al, Cd, Co, Cu, Fe, Mn, Ni, Pb, and Zn by HR-ICPMS (Thermo Element XR). Samples were introduced into the instrument with a peristaltic pump at a flow rate of ~120 μL min^−1^ and passed through an ESI-PC3 Peltier cooled spray chamber before entering the torch. Sample and gas flow rates were optimized for each run; values were 0.75–0.80 ml min^−1^ and 0.20–0.24 ml min^−1^, respectively. Nickel sample and skimmer cones (Spectron) were used. Rhodium was added to each sample as an internal standard for calibrating sensitivity shift of the instrument. Method accuracy and precision were assessed relative to Certified Reference Material CASS5 (Supplemental Table 1). All samples, including operational blanks, were corrected for recovery yield. Average values from the operational blanks were then subtracted from each sample for each time point such that the reported metal concentrations represent only aerosol-derived material.

Measurement of Co in natural seawater samples requires ultraviolet irradiation due to biogenic Co ligands (Saito et al., 2005; Milne et al., 2010; Shelley et al., 2010). Irradiation was not done in this study because the filtered seawater contained no organisms, and the dissolution from dust particles most likely does not create Co ligands, which are biological in origin. Moreover, we have not observed an excess of Co ligands in our speciation studies, and find them to be kinetically inert (non-exchangeable) in surface waters (Saito et al., 2005; Saito and Schneider, 2006) implying that Co-ligand complexes are likely formed intra-cellularly through enzymatic pathways such as the B12 biosynthetic pathway. As a result, the dissolution of Co from dust in this experiment would most likely remain labile and not exchange with the natural cobalt ligand pools.

### Statistical approaches

Two types of statistical tests were included in the analysis of trace metal dissolution behavior for the data shown in **Figure 3**. First, to determine whether dissolution of individual metals were statistically different from each other, an ANOVA was conducted, followed by *t*-tests using the Bonferroni correction to account for multiple comparisons. Second, we calculated 95% confidence intervals for the percent change in dissolved metal content for each metal over time. The 95% confidence interval describes the range of values in which the mean is expected to fall 95% of the time. We used the confidence intervals to comment on the probable dissolution behavior of each metal. For example, confidence intervals that crossed the “0% change” axis indicate that the metal has a high probability of showing instantaneous dissolution behavior. In contrast, confidence intervals falling entirely above the axis are more likely (95% of the time) to show gradual dissolution over time. Importantly, this approach allows metals that are not statistically different in pair wise analysis (such as Co and Zn) to still be classified into separate groups based on their likelihood to dissolve slowly or rapidly.

## Results and discussion

The dissolution experiment was conducted in aged seawater that was 0.2 μm filtered prior to the experiment. Background metal concentration in the water are given in Table 1 and are compared to published concentrations for surface water in the Atlantic Ocean. Al, Cu, Mn, and Ni were all within the expected range (Sohrin and Bruland, 2011), whereas the particle reactive metals Fe and Pb were lower than typical surface concentrations, likely due to sorption onto the walls of the carboy during storage. While close to the expected range, Co levels may be underestimated due to biogenic ligands in the seawater, as the samples were not UV irradiated prior to analysis, as discussed above (Saito et al., 2005; Saito and Schneider, 2006; Milne et al., 2010; Shelley et al., 2010). The metals Cd and Zn were slightly higher than typical surface water concentrations, likely due to low level contamination during the collection process.

### A continuum of aerosol metal dissolution

**Figure 2:**
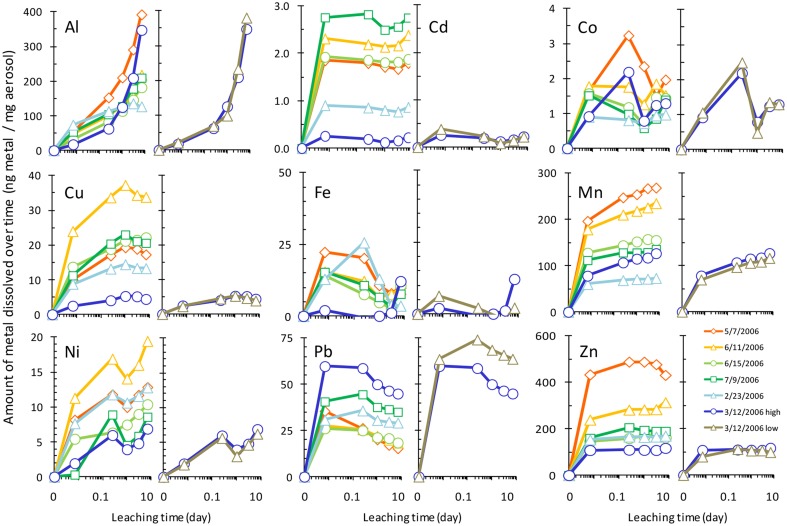
**Time series of aerosol metal dissolution after 10 min, 6 h, 1 day, 3 days, and 7 days exposure to seawater**. Leaching of the sample from 3/12/2006 at two particle to solvent ratios is also shown for each metal (rightmost panels of each pair, denoted by “high” and “low” in legend). Raw data are provided in Supplemental Table 2. Legend in the “Zn” panel shows aerosol collection date.

**Figure 3:**
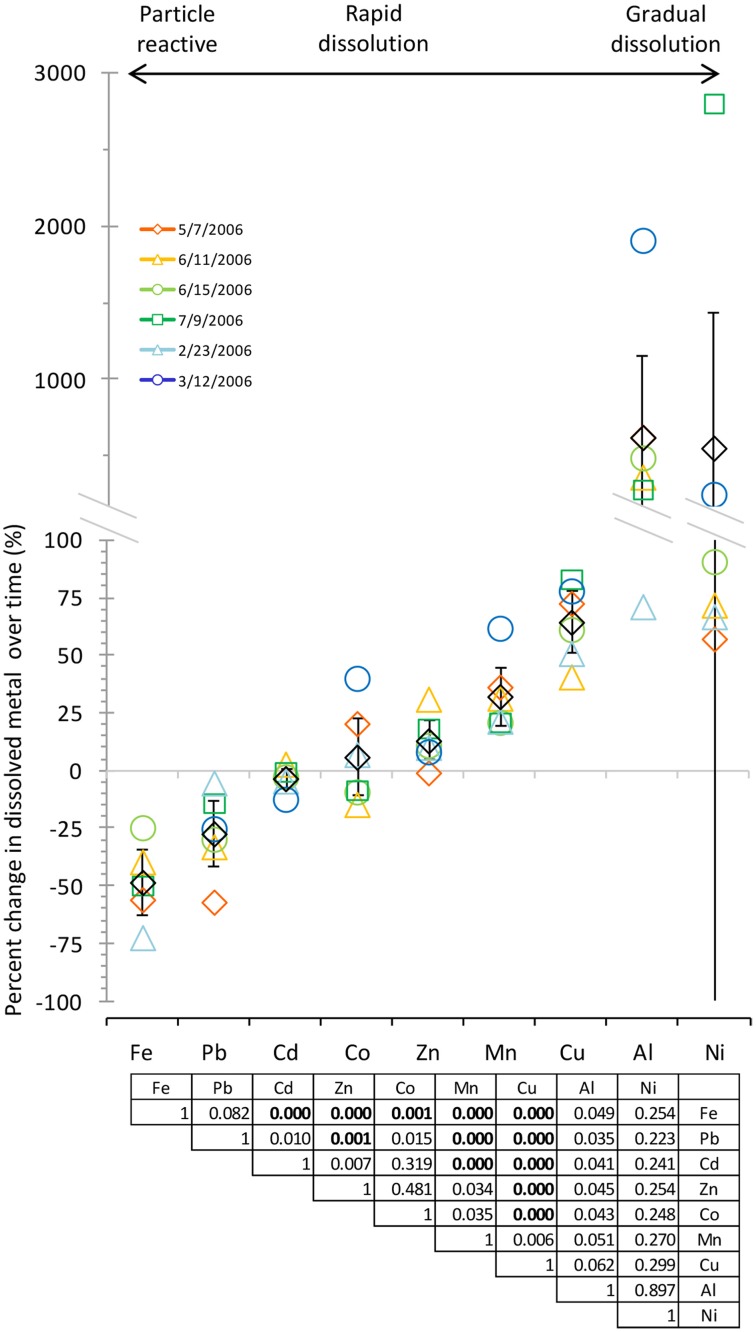
**Comparison of dissolved metal concentrations after 10 min and 7 days of leaching suggested a continuum of behaviors with three modes of dissolution: particle reactive, rapid, and gradual dissolution**. Black diamonds show the average, and error bars show the 95% confidence interval. Metals are arranged from lowest to highest percent change by average. Legend shows aerosol collection date. Statistical analysis via ANOVA shows statistically distinct dissolution behavior between certain metals [*F*_(8, 44)_ = 2.16, *p* = 0.04897]. The table shows *p*-values for *t*-tests between metals using the Bonferroni correction, where *p* < 0.0056 is considered significant (significant comparisons are shown in bold).

The amount of each metal to dissolve from the aerosol samples was measured over time, and Figure 2 shows the dissolution time course with the concentration of each dissolved metal normalized to the mass of aerosol. Metal dissolution behavior occurred along a continuum, where some metals were more strongly influenced by particle reactivity, some dissolved rapidly, and others dissolved gradually over time (Figure 3, Table 3). Pair wise statistical analysis (Figure 3) demonstrates that rather than representing three discrete modes of dissolution, there is overlap between the groups due to variability that likely stems from the chemical and physical characteristics of each aerosol sample.

**Table 3:** **Percent solubility of aerosol trace metals in seawater after 10 min and 7 days of leaching**.

**Metal**	**Total mass of metal per mass of aerosol (mean ± SD)**	**Percent dissolved after 10 min (%)**	**Percent dissolved after 7 days (%)**
Al	11.5 ± 6.1 mg/g	0.45 ± 0.22	2.5 ± 1.0
Cd	2.12 ± 0.79 μg/g	76 ± 31	74 ± 33
Co	9.88 ± 4.33 μg/g	17 ± 10	17 ± 7
Cu	84.7 ± 37.4 μg/g	14 ± 5	22 ± 8
Fe	24.5 ± 10.3 mg/g	0.066 ± 0.039	0.044 ± 0.022
Mn	461 ± 121 μg/g	28 ± 12	38 ± 15
Ni	53.7 ± 28.0 μg/g	12 ± 10	25 ± 13
Pb	90.7 ± 47.6 μg/g	46 ± 15	32 ± 10
Zn	429 ± 287 μg/g	57 ± 24	65 ± 30

To determine which mode of dissolution metals in our samples resembled most closely, we calculated 95% confidence intervals, which show the range of values that the mean has a high (95%) probability falling within (Figure 3). We designated metals with confidence intervals crossing the axis (which indicates no gain or loss in soluble metal content over time) as falling within the “instantaneous dissolution” region of the continuum. These included Cd and Co in our samples, which dissolved rapidly upon seawater exposure. For these metals up to 7 days of extended soaking yielded only small changes in dissolved metal concentration compared to samples measured immediately following seawater exposure (Figure 2).

Metals with confidence intervals falling completely above the axis (Zn, Mn, Cu, and Al in our samples) or completely below the axis (Fe and Pb in our samples) fell closer to the “gradual dissolution” and “particle reactive” ends of the continuum, respectively. While on average the largest percent increase in dissolution after 7 days of soaking was observed for Al (mean = 628%; median = 433%; range = 71–1913%) and Ni (mean = 559%; median = 82%; range = 58–2806), enhanced dissolution over time was also observed for Cu (mean = 65%; median = 67%; range = 41–83%) and Mn (mean = 33%; median = 27%; range = 21–62%). In several samples but not all, Co also suggested a tendency to dissolve gradually over time (Figure 3).

The other end of the continuum includes particle reactive metals for which the dissolved concentrations decreased upon extended soaking in seawater. In our samples these included Fe [mean = −48%; median = −49%; range = −24 – (−72%)] and Pb [mean = −27%; median = −27%; range = −5 – (−57%)]. We did not observe net wall loss for Zn; however, Zn is generally considered a particle reactive metal. The lack of wall loss in our experiment probably occurred due to the background concentrations of Zn in the seawater (Table 1), which may have saturated binding sites on the bottle walls and slowed the sorption of aerosol Zn to these surfaces. Zn may therefore display stronger or weaker particle reactive characteristics depending on the background levels of Zn in the leaching solvent.

We note that the dissolution behavior of many of the metals shows large degree of variability. For example, the confidence interval for Ni, while crossing the axis, also extends far above and below the axis (the confidence interval range for Ni was −324 to 1442%). While all of the actual data points fell above the axis, the large variability in dissolution behavior is skewed by one very high data point, making the confidence interval very large. Other metals had smaller confidence intervals but still had inconsistent dissolution behavior between samples. For example, Co dissolution ranged from samples that were more particle reactive (−14% for the 6/11/2006 sample) to samples that dissolved gradually (40% for the 3/12/2006 sample). Therefore, while on average the dissolution behavior of Co for the samples we tested here tends to be closest to the “instantaneous dissolution” region of the continuum, it is clear that different individual samples may behave differently. The variability would likely be expanded if samples with greater chemical heterogeneity were considered.

**Figure 4:**
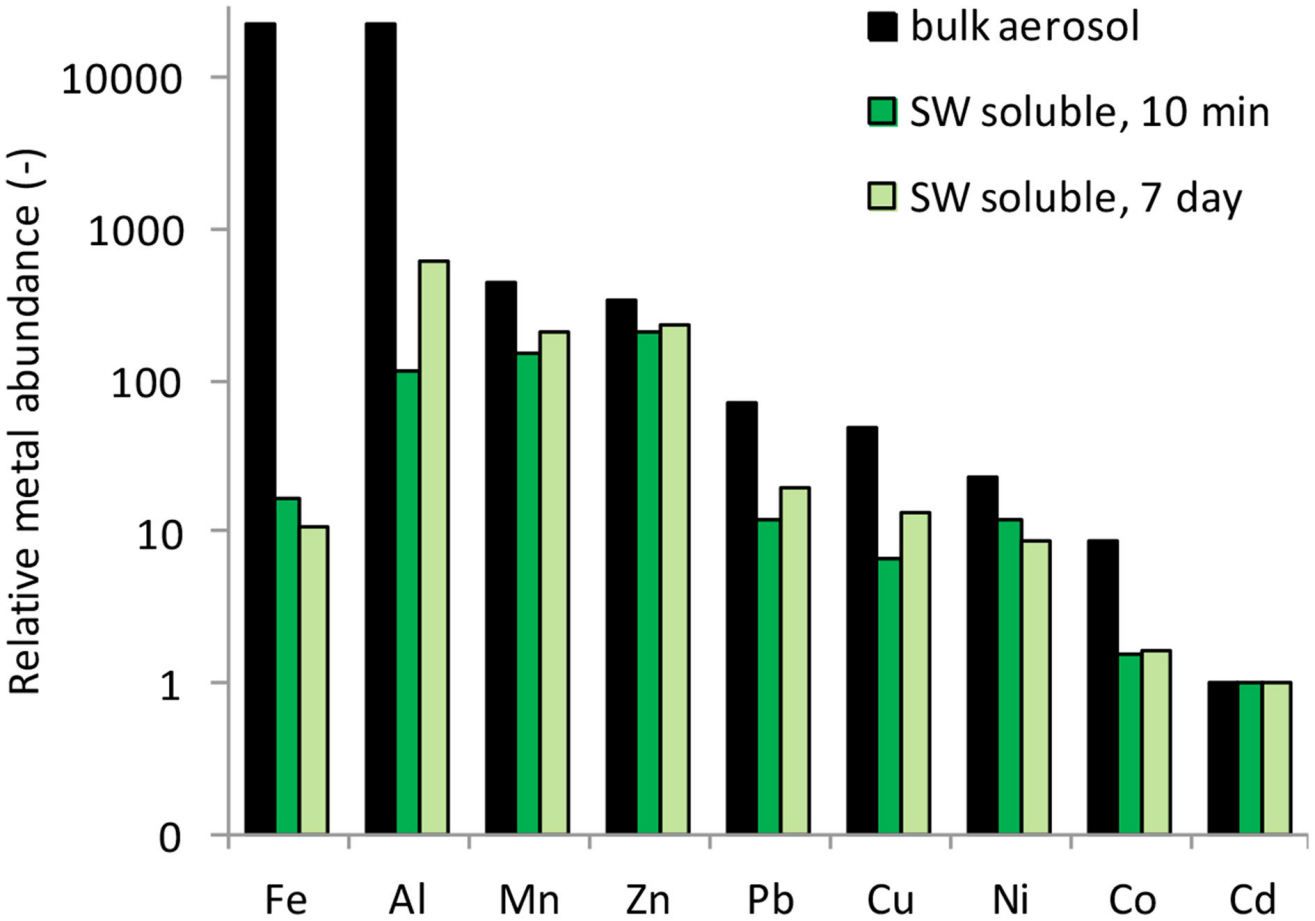
**Elemental molar ratios of metals in the bulk aerosol material, and in the seawater soluble fractions after 10 min and 7 days of leaching**. Metals are arranged from most to least abundant based on the abundance in the bulk aerosol (black bars).

The percent of each metal dissolved relative to the total particulate metal concentrations (e.g., % solubility) is reported for 10 min and 7 day time frames in Table 3. These data fall within the range of seawater solubility previously reported for aerosol samples from this region (Chen et al., 2006). The elemental molar ratio of metals in the bulk aerosols (e.g., the ratio based on total metal content in the aerosol) was 23,189Fe: 22,651Al: 445Mn: 348Zn: 71Cu: 48Ni: 23Pb: 9Co: 1Cd (Figure 4), whereas the ratio of seawater soluble metals after 10 min of leaching was 16Fe: 115Al: 154Mn: 213Zn: 12Cu: 7Ni: 12Pb: 2Co: 1Cd and after 7 days of leaching was 11Fe: 620Al: 205Mn: 240Zn: 20Cu: 14Ni: 9Pb: 2Co: 1Cd. The seawater soluble ratios were calculated based on the relative soluble amount of each metal released into the filtrate, and show that amount of each metal in the soluble fraction differs considerably from that of the total metal content. Because of the large discrepancy between metal ratios in the bulk aerosol compared to the seawater soluble fraction, seawater soluble metal ratios should be reported wherever possible. This will allow models to incorporate aerosol measurements directly, rather than using bulk aerosol metal ratios and assuming percent solubilities, which are sensitive to leaching technique and can vary orders of magnitude depending on the metal in question.

### Implications for leaching protocols and modeling

Many studies have called for a unification of aerosol extraction protocols that would enable data to be compared between different research groups and geographical locations without methodological artifacts (Boyd et al., 2010; Measures et al., 2010; Buck and Paytan, 2012). Solvent, filter type, and duration have all been put forth as variables that should be standardized. Additionally, the relatively high particle to solvent ratio used in this and other aerosol extraction studies differs from conditions in the surface ocean, but is in some cases necessary to bring metal concentrations above detection limit. Boyd et al. (2010) suggest that longer term dissolution could be mimicked by using mineral acid leaching approaches or continuous flow methods to assess maximum Fe dissolution rates such as that described by Simonella et al. (2014). Alternative approaches have also been used to mimic the long term dissolution of Fe from volcanic ash in seawater (Duggen et al., 2007; Olgun et al., 2011) using an electrochemical method to prevent adsorption of the released Fe (Croot and Johansson, 2000).

Performing seawater leaching has the advantage of more closely mimicking the natural environment, but introduces other considerations. The chemical characteristics of seawater vary depending on source, making it difficult to standardize between laboratories. Wu et al. (2007) presented a semi-continuous flow through method for aerosol extraction, and suggest using seawater from the region of interest. This approach mimics characteristics the surface ocean closely, and is especially useful for characterizing metal dissolution over time. However, certain limitations remain; the effects of biological ligand production with time and photochemical redox transformations on aerosol metal solubility that occur in the surface ocean are not captured under laboratory conditions with filtered seawater. Additionally, obtaining sufficient volumes of seawater from regions of interest may not be feasible if the locations are remote, and variability could still be introduced due to seasonal variations in water chemistry. Nevertheless, this study supports using either semi-continuous flow through systems, longer leaching times or stronger solvents for metals such as Al, Cu, Mn, and Ni.

Another factor that can influence aerosol solubility is the particle to solute ratio. For sample 3/12/2006, we repeated the dissolution experiment using two different masses of aerosol added to the same volume of seawater to determine if the particle to solvent ratio influences the extent of dissolution. Dissolution was not strongly affected by the TSP to seawater ratio for most metals considered here; however, a particle concentration effect was observed for Pb (and possibly Fe). For these particle reactive metals, dissolution was higher for smaller doses of aerosol with lower particle to solvent ratios. Approximately 15–20% more Pb dissolved from the sample with lower particle mass (Figure 2). The effect was more difficult to quantify for Fe, where sorption of Fe onto the bottle wall and particles led to very low dissolved Fe concentrations, some of which were below detection limit.

Solubility of metals closer to the particle reactive end of the continuum (Fe and Pb in our samples) would be underestimated using extended leaching procedures in pure water or seawater, but less so for extractions in acidic solution or semi-continuous flow approaches. Wall loss, precipitation, and (re)adsorption of metals like Fe and Pb onto particles, bottle walls, and filters exerts more control over soluble metal content in laboratory leaching experiments than in the surface ocean where the aerosol particle to solvent ratio is lower. Under natural conditions, these metals may show rapid or gradual dissolution patterns that are more similar to the other metals considered here, although the extent of dissolution would be more tightly liked to biota (e.g., ligand production in the case of Fe) and particulate load (sorption onto lithogenic and biological particles in the case of Pb). For these metals, instantaneous leaching methods or semi-continuous flow approaches such as those often used for Fe (Duggen et al., 2007; Olgun et al., 2011) would yield more accurate results than methods with longer leaching times.

Dissolved Al levels in seawater have been used to estimate atmospheric deposition to the ocean on a global scale. Dissolved Al is a robust proxy for atmospheric deposition in most locations, but the model tends to underestimate the amount of deposition in regions with high deposition rates (Measures and Vink, 2000). Factors like mixed layer depth, particle advection and Al solubility all contribute uncertainty to the model, and suggest that less cloud processing of dust in coastal, high-deposition regions could lead to lower solubility of Al (Measures and Vink, 2000) and other metals (Desboeufs et al., 2001, 2003). This study shows that Al dissolution continues up to 7 days after contacting seawater, and supports the idea that dissolution processes in seawater play an important role in determining the total solubility of Al, in addition to clouds. As a result, the residence time of particulate Al in these coastal waters could strongly influence soluble Al levels by affecting time available for dissolution, sinking rate and acidic processing of particles through zooplankton digestion and excretion (Maring and Duce, 1987). Future studies to understand the effects of these processes on Al solubility are timely and could reduce uncertainty in atmospheric deposition models in regions with high deposition.

### Ecological implications

Aerosols can at times provide “complete nutrition” for phytoplankton because they tend to supply macronutrients (N, P) together with metal micronutrients that can co-limit growth. Aerosol N is delivered as nitrate and ammonia, which are immediately soluble, and organic N, of which up to 30% is bioavailable (Peierls and Paerl, 1997). Aerosol P is delivered as phosphate and organic P, and solubility is strongly related to particle origin (anthropogenic vs. crustal sources) (Mahowald et al., 2008; Anderson et al., 2010). Phytoplankton have evolved to take up N and P rapidly when these becomes available, and may perform luxury uptake to fuel growth once the community has depleted the ambient supply (Thingstad et al., 2005; Mackey et al., 2012b). Similar behavior is observed for metals like Fe, which can be stored in ferritin molecules within the cell (Marchetti et al., 2008). However, for other metal micronutrients this approach may not be used, owing to relatively lower biological demand and because metals can be toxic at high doses.

The effect of aerosol metals on phytoplankton growth will depend on the acclimation response time and uptake rate relative to the rate of dissolution. Following a large deposition event, biologically important metals that are rapidly soluble in seawater will be released in a large pulse, favoring species that have high metal demands and intracellular metal storage capacity (e.g., ferritin and similar storage strategies). In the case of Fe which is particle reactive over similar time scales relative to cell growth (Figure 2) and can rapidly precipitate from seawater if ligand levels are low, storage of Fe in ferritin molecules would provide a clear advantage because cells could claim bioavailable Fe before it is lost from the water. In contrast, gradually dissolving metals would be released more slowly over time, favoring sustained growth of species that are more competitive at lower metal concentrations.

Cellular acclimation to increased metal levels may also include the rapid biological production of ligands, which directly influence metal solubility. For example, Fe binding ligand concentrations increased 400% in 1–2 days following moderate Fe enrichment during the IronEx II study (Rue and Bruland, 1997), and Cu binding ligands doubled in response to dust additions in bottle experiments in the Sargasso Sea (Mackey et al., 2012a). In this study the concentrations of gradually dissolving metals increased an additional 33 to over 600% over a seven day period compared to initial amounts. In the open ocean phytoplankton typically double over 1–2 days, suggesting that ligand production rates could rapidly respond to changes in these metal concentrations within sufficient time to alter their solubility. Increased ligand production following atmospheric deposition events could serve to increase the solubility of certain metals and temper the biological responses of organisms by controlling the amount of free metal ions in the water.

The rate of dissolution will also affect toxicity, especially for metals like Cd, Cu, and Ni. Ligand production may limit the toxicity of these elements by regulating the amount of free metal ions in solution. If organisms acclimate rapidly relative to the metal dissolution rate, toxicity may be avoided; this would be more likely for gradually dissolving metals but less likely for rapidly dissolving metals. The solubility of Cu is known to vary considerably between anthropogenic and crustal sources adding another layer of complexity; metals in anthropogenic aerosols have the potential to cause greater toxicity due to their more rapid dissolution and relatively higher total metal content compared to crustal source material (Sholkovitz et al., 2010). Whether aerosol metals cause toxicity will depend on how fast the community can acclimate by producing ligands, the overall uptake rate, the magnitude of the deposition event, the rate of metal dissolution, and the toxicity threshold of the population. Accumulation of metals like Cu and Ni has been observed in aerosol incubation studies following several days of exposure (Mackey et al., 2012a), and likely reflects the combination of low biological demand for and gradual dissolution of these metals over time.

## Conclusion

In this study we investigated the role of extraction time on aerosol trace metal leaching behavior. Dissolution behaviors occurred along a continuum and considerable variability in dissolution exists between samples with different physical and chemical characteristics. Based on the confidence intervals of the dissolution patterns we measured in samples in this study, we identified that metals fell along different regions of the continuum, including rapid dissolution (Cd and Co), gradual dissolution (Zn, Mn, Cu, and Al), and dissolution that is affected by interactions with particles (Fe and Pb). The dissolution kinetics of aerosol metals can influence bioavailable metal micronutrient inventories, and hence phytoplankton growth characteristics, in the surface ocean where particles may remain suspended and leach over a period of days. The different modes of dissolution behavior observed here suggest that to accurately understand and model the effect of atmospheric deposition on phytoplankton communities, the effects of extended leaching on different metals should be taken into consideration.

### Conflict of interest statement

The authors declare that the research was conducted in the absence of any commercial or financial relationships that could be construed as a potential conflict of interest.
