# Supplementary material for: Rapid and gradual modes of aerosol trace metal dissolution in seawater
Source: Front Microbiol. 2015 Jan 21;5:794. doi: 10.3389/fmicb.2014.00794 (PMC4301189; doi:10.3389/fmicb.2014.00794)
Supplement: Supplementary file 1 [file Presentation1.PDF]

Supplemental Table 1: Acidified MQ detection limit and CRM analyses. Units are ng/kg.

Metal	MQ detection limit*	Recovery (%)	CASS5 analyses	
			Measured	Certified
		av	av ± SD	
Al	49.2		487 ± 13	NA
Mn	1.84	82%	2110 ± 86	2560 ± 200
Fe	13.5	105%	1477 ± 39	1400 ± 110
Co	0.99	88%	84.9 ± 3.8	93
Ni	13.15	93%	301 ± 10	322 ± 22
Cu	1.52	100%	371 ± 15	371 ± 28
Zn	184	105%	735 ± 25	702 ± 67
Cd	0.94	116%	24.6 ± 1.5	21.0 ± 1.7
Pb	0.42	79%	8.6 ± 0.4	11 ± 2

* Detection limit calculated by 3×standard deviation of the acidified MQ blanks (4 replicates).

Supplemental Table 2: Seawater soluble aerosol metal concentration data in ng/L. Values have been blank corrected. Values are not operational blank corrected; the concentrations of the operational blanks (Op Blk) are shown in the rightmost columns.

	Time	5/7/06	6/11/06	6/15/06	7/9/06	2/23/06	3/12/06 high	3/12/06 low	Op Blk 1	Op Blk 2
**Al**	10min	2444	1850	1630	1749	3921	1378	1282	880	749
	6h	5303	2912	2886	2484	5432	2719	2199	861	504
	1d	7321	3797	3986	3165	6168	5042	3123	934	977
	3d	9236	3907	4319	3512	6112*	7248	5543	497	350
	7d	12615	5593	5576	4357	6056	12077	9086	667	784
**Cd**	10min	68	63	63	59	50	20	20	12	11
	6h	68	62	63	62	49	20	18	14	12
	1d	66	62	63	58	48	18	16	15	14
	3d	64	62	62	58	46	19	16	13	13
	7d	66	65	62	60	49	19	17	12	12
**Co**	10min	95	85	87	71	83	75	68	67	23
	6h	119	61	53	38	55	93	76	25	18
	1d	84	41	31	23	43	39	23	22	DL
	3d	47	44	41	17	42	43	32	DL	Dl
	7d	61	36	40	26	42	44	30	DL	DL
**Cu**	10min	426	659	489	318	493	204	173	129	118
	6h	634	872	621	473	673	252	218	124	116
	1d	623	871	603	437	644	211	154	40	38
	3d	622	819	623	415	611	220	152	47*	54*
	7d	585	818	654	419	620	207	148	55	70
**Fe**	10min	930	599	633	520	798	320	393	339	DL
	6h	893	553	483	466	1358	263	328	423	DL
	1d	443	264	245	233	662	DL	DL	211	DL

	3d	329	260	DL	DL	246	DL	DL	DL	DL
	7d	243	DL	229	DL	DL	340	DL	DL	DL
**Mn**	10min	6004	4028	3455	1987	2570	2599	1576	51	42
	6h	7556	4753	3894	2281	2934	3545	2182	68	43
	1d	7746	4917	4095	2264	3011	3792	2364	41	37
	3d	8161	5102	4253	2297	3068	3892	2443	56	64
	7d	8178	5284	4172	2389	3110	4177	2594	34	35
**Ni**	10min	688	693	586	447	762	504	480	444	439
	6h	760	780	571	557	897	597	526	400	405
	1d	491	503	388	268	635	318	255	190	189
	3d	536	539	420	282	666	337	283	182	180
	7d	535	581	423	295	683	371	283	143	150
**Pb**	10min	1107	642	727	735	1323	1984	1430	35	27
	6h	826	603	698	803	1535	1949	1663	32	30
	1d	626	508	583	668	1301	1648	1525	18	13
	3d	535	460	569	646	1263	1534	1464	15*	12*
	7d	478	424	504	620	1241	1481	1415	13	12
**Zn**	10min	13840	6032	4584	3477	7158	4223	2444	758	642
	6h	15232	6722	4669	3993	7397	3997	2886	398	452
	1d	15097	6612	4781	3669	7189	3845	2614	339	256
	3d	14748	6610	4939	3572	7236	3807	2605	281*	273*
	7d	13326	7265	4582	3552	7392	4082	2427	224	289

DL = below detection

* = missing data; values for these time points were calculated from the average of the time points before and after.
